# Potential use of clinical polygenic risk scores in psychiatry – ethical implications and communicating high polygenic risk

**DOI:** 10.1186/s13010-019-0073-8

**Published:** 2019-02-27

**Authors:** A. C. Palk, S. Dalvie, J. de Vries, A. R. Martin, D. J. Stein

**Affiliations:** 1 Department of Psychiatry, University of Cape Town, Groote Schuur Hospital, Observatory, Cape Town, 7925 South Africa; 2 Department of Psychiatry and SA MRC Unit on Risk and Resilience in Mental Disorders, University of Cape Town, Groote Schuur Hospital, Observatory, Cape Town, 7925 South Africa; 3 Department of Medicine, University of Cape Town, Groote Schuur Hospital, Observatory, Cape Town, 7925 South Africa; 4 Analytic & Translational Genetics Unit, Massachusetts General Hospital, Boston, MA USA; 5 Stanley Center for Psychiatric Research & Program in Medical and Population Genetics, Broad Institute of MIT and Harvard, Cambridge, MA USA

**Keywords:** Polygenic risk score, Ethics, Bioethics, Psychiatric genetic risk, Risk communication, Risk interpretation, Complex risk

## Abstract

Psychiatric disorders present distinct clinical challenges which are partly attributable to their multifactorial aetiology and the absence of laboratory tests that can be used to confirm diagnosis or predict risk. Psychiatric disorders are highly heritable, but also polygenic, with genetic risk conferred by interactions between thousands of variants of small effect that can be summarized in a polygenic risk score. We discuss four areas in which the use of polygenic risk scores in psychiatric research and clinical contexts could have ethical implications. First, there is concern that clinical use of polygenic risk scores may exacerbate existing health inequities. Second, research findings regarding polygenic risk could be misinterpreted in stigmatising or discriminatory ways. Third, there are concerns associated with testing minors as well as eugenics concerns elicited by prenatal polygenic risk testing. Fourth, potential challenges that could arise with the feedback and interpretation of high polygenic risk for a psychiatric disorder would require consideration. While there would be extensive overlap with the challenges of feeding back genetic findings in general, the potential clinical use of polygenic risk scoring warrants discussion in its own right, given the recency of this possibility. To this end, we discuss how lay interpretations of risk and genetic information could intersect. Consideration of these factors would be necessary for ensuring effective and constructive communication and interpretation of polygenic risk information which, in turn, could have implications for the uptake of any therapeutic recommendations. Recent advances in polygenic risk scoring have major implications for its clinical potential, however, care should be taken to ensure that communication of polygenic risk does not feed into problematic assumptions regarding mental disorders or support reductive interpretations.

## Background

Psychiatric disorders present distinct clinical challenges due to the fact that their diagnosis relies predominantly on observing a patient’s behaviour and on their reporting symptoms rather than on clinical tests for biomarkers. This is mostly attributable to the sheer complexity of psychiatric disorders which are heterogeneous in both aetiology and symptomology. For this reason, establishing evidence of pathophysiological functioning through identifying definitive biomarkers that could assist in more efficient risk identification, diagnosis and prognosis as well as improved treatment of psychiatric disorders has been a major research imperative for a number of decades. Given advances in our understanding of the genetic basis of psychiatric disorders, the question arises whether metrics that describe these, such as the polygenic risk score (PRS), could be used as biomarkers.

PRS is a research tool that is currently used in a range of genetic studies. PRS is calculated by multiplying the number of independent risk alleles an individual carries by the effect size of each variant, then summing these products across variants. While PRS currently lacks predictive power and may never possess clinical utility for certain psychiatric disorders, for disorders with high heritability such as schizophrenia and bipolar disorder, there is a growing possibility that some form of PRS may be developed for the clinical context. It is therefore worthwhile to consider any ethical implications of such a test.

In the first part of this paper we provide an outline of some of the relevant scientific and methodological challenges and introduce PRS. In the second part we discuss four areas in which the use of polygenic risk scores in psychiatric research and clinical contexts could have ethical implications with a particular focus on potential challenges that could arise with the feedback and interpretation of high polygenic risk for a psychiatric disorder. While there would be much overlap with the challenges associated with the feedback of genetic findings in general, we mainly focus on the potential difficulties associated with communicating and interpreting complex genetic risk information. To this end, we look at how lay interpretations of risk and genetic information could intersect. Consideration of these factors would be necessary to ensure effective and constructive communication and interpretation of polygenic risk information which, in turn, could have implications for the uptake of any therapeutic recommendations. Recent advances with PRS have major implications for its clinical potential, however, care should be taken to ensure that interpretation of polygenic risk does not feed into problematic assumptions regarding mental disorders or support reductive interpretations.

### Genetic markers for psychiatric disorders

There is considerable interest in identifying the genetic determinants of psychiatric disorders. Collaborations like the Psychiatric Genetics Consortium (PGC) have played a key role in delineating the role of genetic variants in conferring risk for major psychiatric disorders such as schizophrenia, autism spectrum disorders, bipolar disorder, major depressive disorder and attention deficit and hyperactivity disorder [1]. However, as advances have been made in this area, the sheer complexity of the genetic underpinnings of these disorders has also become increasingly apparent. As is true in the case of many other complex diseases (e.g. diabetes mellitus, hypertension, coronary heart disease and some cancers), the vast majority of psychiatric disorders are highly polygenic, with thousands of independent genetic associations of small effect contributing meaningfully to risk. In contrast, rare monogenic or Mendelian disorders such as Fragile X or Noonan syndrome account for a minority of psychiatric disorders and are caused by single gene mutations. In addition, psychiatric disorders, and complex illnesses in general, are multifactorial; risk is conferred not only by additive genetic effects but also by non-genetic, environmental interactions. Further complexity is due to considerable overlap in the genetic basis of different psychiatric disorders. For example, an individual at risk for developing schizophrenia will also be at risk for bipolar disorder [2]. This overlap presents challenges for the coherence of current psychiatric nosology which, for diagnostic purposes, entails categorising disorders as discrete entities [3].

Despite these challenges, the rapid progress in the field of genetics, and related areas, coupled with greater specificity due to ever-increasing sample sizes, gives cause for optimism that the clinical utility (i.e. the ability to demonstrate “user acceptability and accuracy”, as well as improving “clinical decision making…[and] clinical outcomes” [4]) of genetic markers in psychiatry may be imminent. As our knowledge of the genetic basis of psychiatric disorders develops, it could also support a more targeted therapeutic approach for psychiatric disorders, known as precision medicine (PM) [5]. PM entails tailoring clinical decisions according to an individual’s biological and relevant environmental factors that impact disease outcomes, in order to maximise treatment efficacy and minimise adverse side-effects. This move towards a more personalised approach to treatment has been informed by the major costs associated with suboptimal treatment and adverse drug reactions [6]. While there are a number of factors that contribute to adverse drug reactions, in many cases the genetic profile of the patient is implicated in negative side effects [7].

### Genome-wide association studies and polygenic risk scores

One of the primary ways in which our understanding of complex traits has been expanded over the last decade is through genome-wide association studies (GWAS) and, more recently, through whole exome sequencing studies (WES). Both of these involve experimental designs that explore genetic variation at the population level in order to delineate genetic contributions to disease risk and prediction with the ultimate aim of treating or, if possible, preventing complex diseases [8]. The power of such studies to robustly identify associations between genetic variants and traits, and thus, to accurately predict disease risk depends primarily on sample size [8]. To achieve statistical significance, such studies require large numbers of samples of both cases and controls.^1^

The logistical difficulties involved in obtaining such vast numbers of samples have led to the introduction of meta-analysis, which combines results from smaller studies. To this end, genomics research is frequently conducted in large consortia involving collaboration on an international scale between numerous sites. An example, mentioned above, is the PGC which was created in 2007 with the aim of conducting meta-analyses to deepen existing knowledge of the aetiology of psychiatric disorders. One of their key findings has been the identification of 108 schizophrenia-associated genetic loci, indicating that risk is conferred by thousands of common alleles of small effect [9] . Using data obtained from multiple GWASs, the PGC has also advanced the use of polygenic risk scoring for psychiatric disorders [2].

PRS is a statistical tool that is used in research to predict genetic risk for complex diseases. A PRS can be calculated using summary statistics from a GWAS “discovery” sample in which millions of single-nucleotide polymorphisms (SNPs)^2^ have been scanned in order to identify those alleles that distinguish cases from controls in the particular phenotypic trait or disease that is being studied. The set of SNPs that has been identified in the discovery GWAS generally comprises thousands of risk alleles of small effect. This genomic information from the discovery sample is then used to calculate the PRS of each individual in an independent “target” sample [10]. The most common way of calculating a PRS is to sum the number of risk alleles that an individual possesses multiplied by the trait-specific weight as reported by the discovery dataset [11]. The generated PRS would essentially inform of the degree of genetic risk an individual has for developing the disease in question.

#### Clinical potential

PRS is currently limited to research contexts where it is used for various purposes such as testing treatment modalities and predicting treatment outcomes, testing associations between traits and/or diseases, and determining genetic overlap between disorders (see [12–16]). However, the possibility of adapting PRS for clinical use in psychiatry is something that is now being considered [17–19]. This is not surprising given the polygenicity and heritability of psychiatric disorders as well as the difficulties associated with their diagnosis and treatment, and thus, the dire need for legitimate biomarkers. In fact, PRS may arguably be able to aid differential diagnosis. Recently PRS was able to identify both shared genetic components as well as genetic differences between schizophrenia and bipolar disorder for the first time [20]. In cases where a patient’s symptoms fit multiple disorders, greater diagnostic precision would enable a more accurate understanding of disease course and treatment (see [20] for a full discussion of the implications of this possibility). Indeed, this raises the question of how PRS would be used in a clinical setting.

In order to facilitate understanding and use, PRS is generally converted into a standardised score that follows a normal distribution, with higher PRS corresponding to higher risk [17]. In the clinical context, PRS could be used to determine an individual’s position on this distribution so that those whose scores fall above a sufficiently high, predefined threshold would be informed of this risk. It is unclear how extreme a score would have to be to achieve clinical relevance, however it could be speculated that a PRS in the top 1–5% of the population would warrant feedback [17].

In their brief paper exploring the possibility of translating PRS into a clinical context, Lewis and Vassos discuss potential advantages [17]. First, calculating a PRS is relatively straightforward and requires only a DNA sample. Second, DNA is stable from birth, and as sample sizes in genetic studies increase, PRS will continue to become more accurate. Third, and most importantly, knowing that one is at high risk for developing a disorder well in advance of onset could enable pre-emptive treatment or the avoidance of environmental stressors that could trigger onset, thereby enabling possible prevention or mitigation of the disorder [17].

PRS could, for example, be particularly useful for busy primary care doctors, as a tool to screen patients who are at risk. Help in the early identification of, say, subtle prodromal symptoms of schizophrenia, could ensure that such patients are referred to specialist care more swiftly. The question of when, and how, to treat patients at high risk is a challenging one, given the fact that it is only possible to identify the prodromal period retrospectively, i.e. once the disease has progressed [21]. Pre-emptive treatment of patients with prodromal schizophrenia has, however, been associated with improved clinical outcomes in various studies [22]. An example of such treatment is administration of low doses of antipsychotics (e.g. risperidone) in conjunction with psychotherapy (e.g. cognitive behavioural therapy) [23, 24].

The greater precision of risk identification afforded by PRS could be useful not only at a clinical level, if it is able to improve patient outcomes, but also at a public health level as a means of developing intervention thresholds, or in terms of resource-allocation. The potential of PRS to be used in such a way could be assessed through, for example, an evidence-based initiative offering phase-specific psychosocial treatments to people at very high risk for developing schizophrenia, where high risk is defined by PRS. In this context, determining a specific numeric threshold, above which treatments were associated with better outcomes, would be useful.^3^

#### Challenges to clinical translation

Despite the promise that PRS holds, there are certain technical barriers that currently prevent its clinical translation, the largest of which is discussed next. PRSs are currently able to explain between 1 and 15% of the variation between cases and controls in research contexts [8]. This has been regarded as insufficient predictive ability to allow robust translation into a clinical context [25, 26]. However, the utility of being able to explain 15% of risk for a disorder, in the *entire* population, must also not be underestimated. For an individual at the top end of the risk distribution, the relative risk will be significantly higher than 15% increased risk. Individuals at the top end of the distribution may be at three to five times higher risk than the general population for certain diseases, with even higher relative risk for disorders such as schizophrenia [18]. This information has major relevance from the perspective of prevention and treatment. In fact, it has been argued that PRS is already more useful for identifying a larger patient population at risk for common disorders, than some monogenic tests for rare disorders which are currently used in the clinical context [18].

The limited variation explained by PRS is largely attributable to what has been coined the problem of ‘missing heritability’. A disorder such as schizophrenia is estimated to be approximately 80% heritable, with heritability referring to the proportion of the phenotypic variation that is attributable to genetic variation. However, depending on the measure used, the highest proportion of variation that has thus far been captured by PRS for a psychiatric disorder is 7% on the liability scale for schizophrenia [9]. There are several possibilities regarding these ‘missing genes’ [27]. While it has been estimated that common variants may explain up to half the heritability for numerous common diseases, many common risk variants may have even smaller effects that will only be detected with sufficiently large sample sizes [28]. Furthermore, it has been confirmed that risk is conferred by common and rare (de novo) variants acting additively in the case of autism spectrum disorders [29], this may also be the case for other disorders. There is also the possibility that unknown non-additive^4^ genetic variation could be a component of genetic liability [30]. As GWAS sample sizes increase, the predictive power and efficacy of PRS also increases [8]. However, despite the allure of a tool such as the PRS, its translational potential needs to be empirically evaluated. Furthermore, there are potential ethical concerns regarding the use of PRS in research and clinical contexts.

### Ethical concerns

Genetic counselling for psychiatric disorders is generally limited to cases where there is an established family history of a disorder, such as schizophrenia, or a known risk of dominant or recessive inheritance of diseases associated with intellectual or psychiatric impairment or disability. This is likely to change with increasing public awareness of the strong hereditary component of psychiatric disorders [31] and uptake of direct-to-consumer genetic testing [32]. Research indicates that psychiatric healthcare professionals believe that this would be a positive thing, in terms of the valuable “psychosocial support” [33] that genetic counselling provides [33]. Furthermore, studies indicate that should genetic testing for psychiatric disorders become possible and widely available there would be considerable public uptake, [32, 34], although in some cases support for such hypothetical tests has been dependent on the extent to which they would deliver definitive, as opposed to probable, results [35]. While these studies indicate a hypothetical demand for a test such as the PRS, there are potential ethical concerns with respect to its use that warrant consideration. Here, there would be a broad array of concerns including the possibility that PRS could exacerbate existing health inequities, eugenics concerns regarding prenatal testing and challenges associated with testing of minors, the possibility that such a test could entrench stigmatising or reductive assumptions regarding mental disorders, the potential for discriminatory use, and challenges regarding feedback and interpretation of high polygenic risk. As most of these concerns are associated with genetic testing in general, it is necessary to examine their implications for the use of PRS, in particular. We discuss four areas in which the use of PRS could have ethical implications.

#### The potential exacerbation of health inequities

The majority of GWASs have been conducted in high income countries (HICs), and, even within these contexts, have included mostly participants of European ancestry [36]. The predictive ability of PRS is therefore much higher for these populations. The need to include populations with non-European ancestry in these studies, and in particular, populations with African ancestry, which are significantly underrepresented, has been noted [36–39]. This has become even more pertinent in light of the fact that direct-to-consumer-genetic companies are poised to offer PRS testing for certain diseases with predictive ability that is avowedly “race-restricted” [40].

There are several reasons that warrant greater representation of populations of African ancestry in GWASs. Given that humanity originated in Africa, such studies may provide valuable insights regarding missing gaps in our knowledge of human evolutionary history in general [41]. In addition, genomes of African ancestry are characterised by significant levels of genetic diversity and unique genetic variants, due to patterns of migration and admixture [39]. Studying the genomes of populations of African ancestry therefore holds major potential for deepening our understanding of the genetic basis of various complex diseases and traits [36]. Furthermore, and most importantly, because PRS has the potential to enhance clinical outcomes, the fact that its predictive ability is limited for populations of non-European ancestry represents an injustice. In fact it has been argued that this constitutes the most serious ethical challenge facing the translation of PRS into the clinical context [37]. Martin et al. have also discussed various systemic challenges that have informed the neglect of diversity in genetic studies and provide suggestions to address this [36]. Initiatives such as Human Heredity and Health in Africa (H3Africa) and Neuropsychiatric Genetics in African Populations (Neuro-GAP) will be of great significance in the move for greater global health equity [39].

#### The misinterpretation of findings and potential for stigma and discrimination

A second concern relates to implications associated with how PRS is currently used. In research contexts PRSs have been calculated for a number of complex behaviours and traits as well as to test correlations between traits. While there are tools that are more appropriate for such purposes, PRSs have, for example, been used to test genetic overlap between psychotic disorders, addiction [42] and substance use [43] and even between psychosis and creativity [44]. They have also been used to predict alcohol use [45] and dependence [46], antisocial behaviours [47], intelligence [48], educational attainment [49] and to test correlations between genetic risk for low educational attainment and criminal behaviour [50]. The main underlying concern in all these examples is the potential for misinterpretation of such findings. In particular, the way in which this kind of information is made more accessible to the public is crucial. The dissemination of information regarding progress in health-related fields such as genetics has grown considerably due to the ease of access to online information. However, the process of translation frequently involves simplifying or exaggerating information so as to capture attention [51, 52]. Without the requisite nuance in explanation and understanding, this information is easily misinterpreted. In the case of the correlations that are currently being tested, the concern would be that misinterpretations could exacerbate stigmatising assumptions regarding mental disorders, or that this information could be used for discriminatory purposes. While certain countries have legislation that offers protection against the discriminatory use of genetic information, such as the Genetic Information Nondiscrimination Act (GINA) in the United States, this is not the case in numerous other countries. Furthermore, GINA has been criticised on account of the fact that the protection it offers is limited to preventing discrimination in employment and health insurance; it does not apply to other forms of insurance, small companies (with fewer than 15 employees), or various other areas [53].

In terms of a concern for an increase in stigma, studies indicate that biogenetic explanations may be associated with “lower social acceptance” [54] in the case of certain mental disorders or with other negative connotations [55–58]. This may be attributable to the tendency of biogenetic explanations to elicit various reductive, determinist or essentialist interpretations. For example, where complex behaviours are shown to have genetic determinants this could result in interpretations in which the role of genetic factors in behaviour and health is overestimated at the expense of social determinants, a concern that has been discussed extensively in the ethics literature ([59–61], in particular, see [62] for a discussion of this as it pertains to PRS specifically).

In some cases, biogenetic explanations are associated with more tolerant attitudes towards certain behaviours [54, 63], however, it is important to examine why this is so. While an increase in tolerant attitudes is a positive outcome, if tolerant attitudes are informed by the perception that biological causal attributions decrease or eradicate agency in some way, this would be indicative of an underlying deterministic assumption which may have unanticipated consequences. Furthermore, tolerance that is informed by a perception of genetic causation also indicates the operation of the naturalistic fallacy [60]. This refers to the process of deriving normative conclusions from natural states of affairs, or, deriving an ‘ought from an is’. While this would be an example of an essentialist belief that happens to be supporting a positive outcome, it is not without risk. As pointed out by Dar-Nimrod, political sentiments are subject to change, and thus, favourable causal attributions that currently act as protective mechanisms may also change [60]. We discuss the issue of determinism further in the next section.

#### PRS testing of minors and eugenics concerns regarding prenatal testing

A third area of concern would be the use of PRS for various forms of prenatal testing or the testing of minors. In the latter case, parents may wish to ascertain their child’s PRS for a particular disorder, especially when there is a family history. There would be compelling reasons for doing so, given strong evidence of association between various environmental factors in childhood and adolescence, and disorders such as schizophrenia, bipolar disorder and depression [64, 65]. While some of the childhood environmental risk factors for developing schizophrenia that have been identified would be impossible for some families to avoid (e.g. urbanicity and poverty), and others should be prevented regardless (e.g. maltreatment and bullying), there are certain avoidable risk factors that increase vulnerability such as use of cannabis and stimulants in adolescence [64].

The ethical permissibility of genetic testing of minors has been addressed extensively [66] and studies have looked at how knowledge of genetic risk affects the self-conception of adolescents [67]. In particular, the ethical considerations and benefits of psychiatric genetic counselling for adolescents have also been discussed [68]. However, it must be noted that genetic counselling does not require genetic testing [68], therefore, ongoing discussion and studies should focus on how psychiatric genetic counselling for minors could be impacted by the possibility of being accompanied by polygenic testing. While there is undoubtedly much overlap with the ethical issues related to the genetic testing of minors in general, psychiatric PRS testing arguably intensifies these concerns due to the fact that is likely that the disorders it would mostly be used to predict risk for, would be those with the highest heritability, such as schizophrenia and bipolar disorder, both of which are subject to high levels of stigmatisation [69]. Adolescents receiving feedback of high PRS for such disorders may be at particularly high risk for internalized stigma and potentially detrimental effects associated with negative self-labelling [70, 71].

The potential use of PRS for various forms of prenatal testing, including preimplantation genetic diagnosis (PGD), presents distinct ethical concerns. PGD has been used for a number of decades to screen embryos created through in vitro fertilization (IVF) for various incurable monogenic diseases, such as cystic fibrosis, Huntington’s disease and Tay-Sachs, and more controversially for chromosomal disorders such as trisomy 21 (Down syndrome) [72]. PGD has generally been regarded as ethically preferable to prenatal testing as it avoids the dilemma of termination of pregnancy [73]. However, a concern with PGD is its potential to be used for eugenics purposes [74, 75]. In this regard, PRS is now being marketed in the commercial sector as a means of testing embryos generated through IVF for ‘intelligence’, through screening out those embryos at risk for mental disorders [76]. Given the fact that PRSs can be calculated for the traits discussed above, there is major concern that its marketing by direct-to-consumer-genetic companies in this way will heighten intolerance of diversity and increase stigma towards mental disorders, permitting PRS to be used for eugenics purposes. In addition, it must be noted that clinical genetic testing is generally highly quality controlled – in the United States for example, it is performed by CLIA-certified laboratories – and is likely to be accompanied by a referral to a trained genetic counsellor. Direct-to-consumer testing laboratories have been criticised for not having the same data quality and accuracy and for a lack of transparency regarding the techniques they use [77, 78]. They may also not have access to professionals who can assist in data interpretation [79].

In the remainder of this paper, we focus on what we consider would be the most likely and widespread application of a clinical PRS: cases in which a consenting adult patient has submitted to PRS testing for screening purposes. In particular, we explore the challenges associated with feedback of high polygenic risk for developing a psychotic disorder such as schizophrenia or bipolar disorder. Here, there would be significant overlap with the ethical challenges associated with the feedback of genetic findings in general [80]. There has been abundant research and discussion of the nature of these challenges which include: issues of privacy and confidentiality, implications for family members, the potential for stigma, and the way in which such information is communicated and understood, so as to minimize psychological distress to patients [81–83].

While all of these concerns would be relevant in the case of a clinical PRS, we argue that particular attention should be paid to the difficulties associated with the communication and interpretation of results. This would be due, in part, to the fact that, given the etiological complexity of psychiatric disorders, a PRS in the top percentile would be an indicator of risk, not a definitive prognosis. For this reason, nuance and skill would be required in articulating and ensuring correct understanding (both of counsellors and patients) of ‘complex’ risk. While the difficulties associated with feedback of complex genetic risk are not necessarily unique to PRS, they nevertheless warrant consideration given its recency [18]. In the final section that follows we discuss factors regarding the interpretation of both complex risk and genetic information that could pose challenges for PRS feedback.

#### Challenges of feedback of polygenic risk

The concept of risk has a variety of informal and technical definitions. Risk is generally associated with the *possibility of some negative or undesirable event occurring*, or, as *the cause attributed to a negative event*. In this common usage, risk is mostly interpreted according to a personal or subjective framework. For example, while most individuals know that driving poses a risk or that there is a risk of contracting cancer, if pressed to quantify these risks more precisely, estimates will vary widely and generally not accord with the objective or statistical risk concerning the phenomenon in question [84]. In fact, studies indicate low levels of understanding of statistical or numerical risk information not only in the public arena [85] but also in the case of medical professionals [86]. It is therefore likely that quantitative or objective risk will not be interpreted in a predictable or uniform manner. In addition, the difficulties related to the comprehension and interpretation of genetic information in general [87–89] as well as the challenges related to communicating complex genetic risk information have been discussed extensively [90–92]. The comprehension of polygenic risk thus represents an intersection between various constructs that are, understandably, easily misinterpreted due to their complexity. However, if polygenic risk communication is considered similar in kind to communication of other risk indicators in medicine then there are numerous strategies and resources that may be utilised [93].

Considering these factors is important because the aim of communicating a high PRS for a psychiatric disorder would be to prevent onset or mitigate severity, if possible. The foremost challenge would therefore be how best to communicate a high PRS so as to facilitate the uptake of any therapeutic recommendations or requisite preventative measures. This challenge would be even more pertinent in light of studies suggesting that knowledge of personal genetic risk for various common diseases is not necessarily associated with increases in motivation to implement behavioural or lifestyle changes [94–96]. However, the low levels of motivation in such cases may be attributable to low perceptions of threat [97]. As pointed out by Sanderson et al., protection motivation theory (PMT) predicts that if the level of threat is perceived to be sufficiently high and amenable to reduction, this will increase motivation to implement requisite behavioural changes [98]. Feedback of sufficiently high polygenic risk may therefore be an effective motivator for uptake of therapeutic recommendations. These observations aside, it will be important to ensure that PRS feedback is accompanied by meaningful, evidence-based intervention recommendations. Empirical studies of PRS thresholds, such as the example mentioned above, could be helpful in this regard.

An additional factor that warrants consideration is that risk is a normative concept; it is used only to refer to a possible negative event that we seek to avoid. In other words, the notion of risk is directive; there is always some instrumental purpose for seeking risk information or wishing to provide it [99]. We seek risk information so as to mitigate or eradicate this risk, if possible; however, risk, as such, is unavoidable. While there are many risks that we can mitigate, thus giving us a sense of subjective control, there will always be some level of risk that is impervious to our control. In a medical context, there are areas where a certain level of control can be exercised in mitigating risk [100]. Individuals at risk for contracting type 2 diabetes, for example, are able to lessen this risk through behavioural modifications, such as changing their diets, losing weight or exercising. However, perceived subjective control over other forms of medical risk, such as genetic risk, may be drastically reduced because while there are interventions that can reduce overall risk of disease outcomes, the level of genetic risk itself remains relatively stable.

In the case of PRS feedback, it would be important to ensure that information about the stable character of complex genetic risk does not support reductive interpretations. As mentioned in the previous section, such interpretations may result in deterministic assumptions whereby the role played by genes in health and disease is overemphasised at the expense of the crucial role played by environmental and non-genetic factors [101]. This would be counter-productive to the purpose of having communicated a high PRS. Studies of public interpretations of genetic information have produced conflicting results that indicate the presence of both high and low levels of genetic determinism [102]. However, deterministic beliefs are complex and difficult to measure [103]. In addition such beliefs are informed by contextual factors such as religiosity and various social and cultural influences, and are therefore highly variable [104, 105]. On the one hand, an increase in public knowledge of the role played by genetic factors in psychiatric disorders is frequently associated with concomitant determinist and essentialist misinterpretations [59]. Deterministic beliefs, in turn, are frequently coterminous with a sense of fatalism, decreased agency, or being ‘at the mercy of one’s genes’ or biology [106]. On the other hand, studies also indicate the presence of relatively neutral or balanced causal attributions in certain groups [107, 108]. In a study of laypersons’ understandings of health outcomes, Condit et al. observed ‘rampant’ inconsistences in participants’ responses [102]. They hypothesised that these conflicting results may be attributable to the fact that individuals have internalised two distinct and dissonant ‘discourse tracks’ or ways of explaining health and disease: one of ‘genetic causation’ and one of ‘behavioural causation’ [102]. It is presumed that these discourses are encoded in neural networks that develop distinctly, and thus, that they do not operate mutually. This hypothesis has been supported by further research findings [108]. These findings have implications for the framing and communication of PRS information as these tracks may be stimulated by various contextual cues [102]. An appropriate way forward may be to focus on interventions that could effectively connect these two tracks rather than attempting to ‘adjust’ them separately.

Our discussion of some of the factors that require consideration in communicating polygenic risk is by no means exhaustive. Our aim is primarily to make the case that if PRS is ever utilised in a clinical context, research regarding effective communication would be a prerequisite in order to encourage constructive interpretation. Such research should focus on two challenges. Firstly, how to ensure that the relevant healthcare practitioners who would be in a position to order PRS testing and those who deliver PRS feedback have a clear understanding of PRS itself. The acceptance and understanding of PRS by healthcare professionals would be critical to its uptake and dissemination. It would therefore be necessary to ensure that they receive the relevant training that would enable them to ascertain when ordering PRS testing for a patient is warranted. Given the general shortage of genetic counsellors, it is likely that PRS feedback would be delivered by practitioners who do not have expertise in genetics. It would therefore be necessary to equip practitioners with the relevant technical knowledge, including the potential for misinterpretation, and to have a subsequent means of assessing their comprehension.

Secondly, it would be necessary to explore how to translate PRS findings into a more accessible format for feedback that does not lead to misleading oversimplifications and to test the efficacy of these formulations. There are various psychometric tools that have been developed and used to assess genetics literacy in different contexts [103, 109, 110] as well as research that has identified problem areas in genomics, genetics and numeric literacy [111]. Further research that could adapt these tools and findings to devise an instrument relevant for the assessment of understanding of PRS before and after it has been communicated would be valuable. A recent study that assessed the comprehension of psychiatric genomics information of patients with schizophrenia and controls, found that an iterative learning approach led to further improvements in understanding [112]. Iterative learning is a dynamic form of learning that takes the form of a positive feedback loop. Information is presented and explained, after which the ‘student’ is asked to explain this information in their own words, demonstrating their level of understanding. Problem areas are then identified and discussed after which the information is reiterated by the student, and so on. While this study examined iterative learning in conjunction with a particular instrument developed to assess decisional capacity for research participation,^5^ should a clinical PRS become feasible, it would be worthwhile to investigate the adaptability and efficacy of this approach. Research indicates that the iterative approach, also described as “tell back-collaborative inquiry” is “significantly preferred” by patients in demonstrating their understanding, in comparison with other approaches, such as yes-no responses, to questioning [113].

While we have focused primarily on the implications of potential clinical use of PRS for psychiatric disorders, our discussion is relevant to clinical use of PRS for complex (non-psychiatric) disorders in general. However, we posit that feedback of a high PRS for a psychiatric disorder could pose distinct challenges that warrant further attention. For example, there is growing interest in the way in which genetic risk is assimilated into an individual’s “sense of self” [114] or personal identity. We suggest that further discussion should focus on whether the factors discussed above could intersect with stigmatising perceptions of mental disorders to contribute towards a “negative ‘risk identity’” [115].

Furthermore, it is not only the individuals receiving PRS feedback who could be negatively impacted, the issue of ‘associative stigma’ , whereby family members or those with close ties to persons with psychiatric disorders are subject to stigmatising ascriptions, is also a concern [116] As discussed above, given that there is evidence that biogenetic explanations are associated with stigmatising assumptions [55, 117], there is a risk that knowledge of the polygenic heritability of psychiatric disorders could further heighten associative stigma towards family members of persons with psychiatric disorders. While stigmatising assumptions are not unique to psychiatric disorders, the stigma associated with mental disorders is particularly acute and has been recognised by the World Health Organization (WHO) as producing negative impacts in virtually every aspect of the lives of persons living with such disorders, including posing the most significant obstacle to accessing treatment [118]. It is therefore possible that if feedback of high psychiatric risk is interpreted through a stigmatising ‘lens’ this could further confound matters and negatively impact self-conception.

## Conclusion

In this paper we have looked at some of the ethical implications of PRS with a focus on certain challenges that could arise in the communication and interpretation of a high PRS. We take the identified challenges to be a relevant component of an initial exploratory discussion of the clinical efficacy of PRS. This is because the way in which PRS feedback is interpreted would have direct bearing on the uptake of any therapeutic recommendations or preventative measures. Despite the challenges that we have discussed in this paper, we contend that insofar as PRS could assist in more effectively diagnosing, treating or, ultimately, preventing the onset of particular psychiatric disorders, evidence-based clinical translation would be a decidedly positive outcome.

The WHO estimates that “mental disorders [are] among the leading causes of ill-health and disability worldwide” [119]. More specifically, it has been estimated that 7.4% of the global disease-burden is attributable to mental disorders and substance use disorders [120]. Furthermore, meta-analysis reveals that psychiatric disorders are among the leading causes of death; with estimations of 14.3% (roughly 8 million) of all deaths per annum ascribed to psychiatric disorders [121]. Given the enormity of this burden, and the way in which psychiatric disorders tend to negatively impact the lives of individuals and their families, there is arguably a moral obligation to inform individuals who are at particularly high risk so that all possible pre-emptive measures may be taken. There is also a moral obligation to continue to further our knowledge of the aetiology of such disorders in order to continue to improve our responses to them. However, the ethical challenges that will continue to be elicited by the practical applications of this knowledge will require ongoing scrutiny so as to minimise unanticipated and anticipated harms and maximise potential benefits. This paper serves as a point of departure for further discussion of the ethical challenges that could arise through the potential use of clinical PRSs in psychiatry.

## Abbreviations

CLIA: Clinical Laboratory Improvement Amendments
DNA: Deoxyribonucleic acid
GWAS: Genome wide association study
HICs: High Income Countries
IVF: In vitro fertilization
PGC: Psychiatric Genetics Consortium
PGD: Preimplantation genetic diagnosis
PM: Precision medicine
PMT: Protection Motivation Theory
PRS: Polygenic risk score
SNP: Single nucleotide polymorphism
UBACC: University of California, San Diego Brief Assessment of Capacity to Consent
WES: Whole exome sequencing
WHO: World Health Organization

## Footnotes

1 A high level of statistical power is required to avoid false positives and false negatives [122]. An allele with a relative risk of 0–2 is considered to be of small effect. An allele with a relative risk lower than 1.20 requires a sample size greater than 8000 cases (plus controls) to ensure statistical power [123].

2 SNPs which are the most common form of allelic variation, are differences in DNA sequences.

3 We would like to thank an anonymous reviewer for this suggestion and example.

4 Non-additive genetic variation refers to interactions between genes in which the effect produced is more than the sum total of the individual contributions. This is contrasted with additive genetic variation whereby the contribution of both variants is simply the sum of each variant’s effect.

5 This study assessed the ability of iterative learning to improve understanding in conjunction with the University of California, San Diego Brief Assessment of Capacity to Consent (UBACC). The UBACC is a tool specifically designed to assess the decisional capacity of participants who may have impairments in this regard, as well as to improve their understanding by identifying aspects of the research that have not been accurately understood [124].
